# Comparison between chloral hydrate and propofol-ketamine as sedation regimens for pediatric auditory brainstem response testing^☆^

**DOI:** 10.1016/j.bjorl.2017.10.003

**Published:** 2017-10-28

**Authors:** Kamal Abulebda, Vinit J. Patel, Sheikh S. Ahmed, Alvaro J. Tori, Riad Lutfi, Samer Abu-Sultaneh

**Affiliations:** Indiana University Health, Indiana University School of Medicine and Riley Hospital for Children, Department of Pediatrics, Section of Pediatric Critical Care Medicine, Indianapolis, United States

**Keywords:** Procedural sedation, Chloral hydrate, Propofol, Auditory brainstem response testing, Sedação para procedimentos, Hidrato de cloral, Propofol, Potencial evocado auditivo de tronco encefálico

## Abstract

**Introduction:**

The use of diagnostic auditory brainstem response testing under sedation is currently the “gold standard” in infants and young children who are not developmentally capable of completing the test.

**Objective:**

The aim of the study is to compare a propofol-ketamine regimen to an oral chloral hydrate regimen for sedating children undergoing auditory brainstem response testing.

**Methods:**

Patients between 4 months and 6 years who required sedation for auditory brainstem response testing were included in this retrospective study. Drugs doses, adverse effects, sedation times, and the effectiveness of the sedative regimens were reviewed.

**Results:**

73 patients underwent oral chloral hydrate sedation, while 117 received propofol-ketamine sedation. 12% of the patients in the chloral hydrate group failed to achieve desired sedation level. The average procedure, recovery and total nursing times were significantly lower in the propofol-ketamine group. Propofol-ketamine group experienced higher incidence of transient hypoxemia.

**Conclusion:**

Both sedation regimens can be successfully used for sedating children undergoing auditory brainstem response testing. While deep sedation using propofol-ketamine regimen offers more efficiency than moderate sedation using chloral hydrate, it does carry a higher incidence of transient hypoxemia, which warrants the use of a highly skilled team trained in pediatric cardio-respiratory monitoring and airway management.

## Resumo

**Introdução:**

O uso de testes diagnósticos de potencial evocado auditivo de tronco encefálico sob sedação é atualmente o padrão-ouro em lactentes e crianças pequenas que não têm desenvolvimento suficiente para realizar o exame.

**Objetivo:**

O objetivo do estudo foi comparar a sedação de crianças submetidas a testes de potencial evocado auditivo de tronco encefálico com propofol-quetamina e com hidrato de cloral por via oral.

**Método:**

Pacientes entre 4 meses e 6 anos de idade que necessitaram de sedação para a realização do potencial evocado auditivo de tronco encefálico foram incluídos nesse estudo retrospectivo. Foram revisadas as doses dos medicamentos, os efeitos adversos, os tempos de sedação e a eficácia das formas de sedação.

**Resultados:**

73 pacientes foram submetidos à sedação oral com hidrato de cloral, enquanto 117 receberam sedação com propofol-quetamina; 12% dos pacientes do grupo hidrato de cloral não alcançaram o nível desejado de sedação. Os tempos médios de procedimento, recuperação e o tempo total de cuidados de enfermagem foram significativamente menores no grupo propofol-quetamina, entretanto este grupo experimentou maior incidência de hipoxemia transitória.

**Conclusão:**

Ambos os regimes de sedação podem ser utilizados com sucesso para sedar crianças para realização do exame de potencial evocado de tronco encefálico. Embora a sedação profunda com propofol e quetamina ofereça mais eficiência do que a sedação moderada com hidrato de cloral, ela apresenta maior incidência de hipoxemia transitória, o que requer uma equipe altamente qualificada, treinada em monitoramento cardiorrespiratório pediátrico e manejo de vias aéreas.

## Introduction

Hearing loss can lead to significant developmental impairment and speech delay in infants and young children, which necessitates early identification and therapy. An Auditory Brainstem Response (ABR) is an objective method of testing the auditory pathway.^1^ It has been used as a valuable screening test for hearing loss in infants and young children due to their age and development skills.^2^ Although ABR testing is not painful, pediatric patients often require sedation to obtain accurate results. Sedating pediatric patients for ABR could be done either by the anesthesiologist providing general anesthetic or under moderate to deep sedation administered by a procedural sedation service team.^3^

Since children are routinely discharged home after the intervention, the ideal sedative agent would have a rapid onset and favorable side effect profile while producing a sufficient level of sedation for study completion, allow rapid patient recovery, and have a low cost.^3^ Different sedative agents and routes of administration have been utilized for ABR testing such as oral chloral hydrate, intranasal dexmedetomidine, rectal pentobarbital, intravenous propofol and general anesthesia.^4^ Chloral hydrate (CH) was one of the most widely used regimens.^3^, ^4^, ^5^ While its mechanism of action is still unknown, it is believed that its sedative effect is mediated by the Gamma Aminobutyric Acid-A receptors (GABA). Despite the widespread use of CH, serious concerns have been raised about its safety profile.^6^ Additionally, CH has been in short supply since 2013 after manufacturing was discontinued in the United States due to limited availability and low utilization market.

Propofol is an intravenous sedative-hypnotic agent that is used for induction and maintenance of deep sedation and general anesthesia.^7^ Propofol has many properties including a rapid onset, a short duration of action with rapid recovery time and minimal adverse events, which makes it an ideal agent for pediatric sedation in the outpatient setting. The combination of propofol and ketamine for pediatric sedation had been reported to provide optimal hemodynamic stability and reduced adverse effects when compared to propofol alone.^8^ Additionally, the combination of propofol and ketamine had been shown to be beneficial in other medical fields because of allowing lower doses of propofol, resulting in the reduction of the undesirable adverse effects. Many authors reported the advantages of propofol-ketamine combination in terms of hemodynamic profile and pain control in cancer patients undergoing painful procedures.^9^ Emerging data support the safety and efficacy of using propofol outside the operating room for pediatric outpatient procedures and interventions by qualified physicians trained in sedation and advanced airway management.^10^, ^11^, ^12^ Additionally, with increasing numbers of pediatric patients undergoing diagnostic ABR coupled with the relative shortage of anesthesiologists and operating room availability, other pediatric subspecialists, such as pediatric critical care physicians, have stepped in to provide pediatric procedural sedation.^13^

The aim of this study is to compare the efficacy, efficiency and safety of a propofol infusion combined with ketamine to chloral hydrate as a sedative regimen for children undergoing ABR testing.

## Methods

The institutional review board of our institution (Study n° 1204008435R003) approved this retrospective study. All pediatric patients between the ages of 4 months to 6 years old undergoing sedation for ABR were included. Patients less than 5 kg, patients who had a history of a previous failed procedural sedation and patients with cardiac disease were excluded from the analysis. An ABR technician performed all ABR testing for children at our Children's Hospital. The technicians’ team members remained the same during the study period. The study was designed as a retrospective review where patients were analyzed based on sedative regimen used to complete the ABR test.

History and physical exam were performed and documented according to the American Academy of Pediatrics (AAP) guidelines for sedation.^14^ Written consent was obtained from the parent or guardian prior to the procedure. Sedation in the PK group was performed by a sedation team that consisted of a pediatric intensivist and a sedation nurse with a pediatric critical care background who monitored the patient during and after each procedure along with the intensivist in all the cases. CH group sedation was provided by the ABR team that consisted of a pediatric nurse with experience in administering and monitoring patients during moderate sedation. Guidelines for both sedation regimens have been laid down by the AAP regarding the monitoring, management and discharging of children during procedural sedation.^15^ All patients were either classified as ASA-OS I or II per the American Society of Anesthesiologists-Physical Status classification system. Patients were without any solids or formula intake for at least 6 h and 2 h for clear liquids prior to the procedure. Patients in the PK group had an intravenous catheter placed by the sedation team. Physiologic parameters such as heart rate, respiratory rate, oxygen saturation and respiratory plethysmography were continuously monitored. Noninvasive blood pressure were measured every 5 min throughout the procedure and every 15 min after its completion until the patient was fully awake.

For the PK group, a small dose of intravenous ketamine (0.5 mg/kg for patients who weigh less than 20 kg and 0.25 mg/kg for patient who weigh more than 20 kg) was administered followed by intravenous propofol. Propofol was administered as an initial bolus of 1–2 mg/kg followed by an infusion drip of 83 mcg/kg/minute until the end of the procedure. Additional boluses of 1 mg/kg of propofol were given as needed to achieve deep sedation level (level 4) based on the Ramsay Sedation Scale. For those in the CH group, sedation started with 30 mg/kg of oral chloral hydrate and was followed by small subsequent doses (20 mg/kg) within 20 min interval to maximum dose of 1 g if needed to achieve a moderate sedation level. If the child was not sedated during the testing despite the additional doses of the drug, it was considered a failure of sedation.

Adverse events were recorded including development of transient hypoxemia (oxygen saturation of less than 90% for 30 s), hypotension (drop in systolic blood pressure below expected age or dropping by 20% from starting systolic blood pressure), apnea requiring bag-mask ventilation and failure to complete the procedure. Serious adverse events such endotracheal intubation and cardiac arrest were also recorded. Procedure time (PT) was defined as the time between the first doses of sedation until the ABR was completed. Recovery time (RT) was defined as the interval between the completions of the procedure until the patient's level of conscious returned to baseline. Nurse time (NT) was defined as the total time spent by the sedation nurse during the whole process starting from patient arrival to the sedation suite till discharge home.

### Statistical analysis

Comparisons between the PK and CH groups were performed using Wilcoxon rank sum tests for continuous variables and Chi-square tests for categorical variables. Because of the age and weight differences between groups, additional analyses were performed to compare the groups while adjusting for age and weight.

## Results

**Figure 1:**
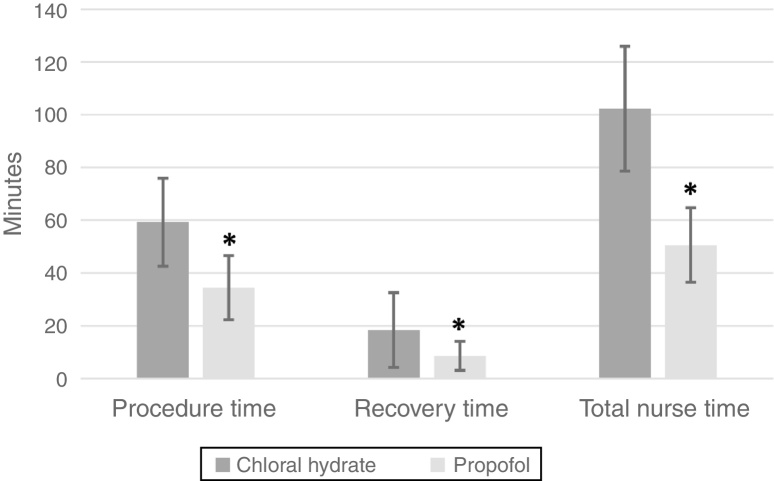
Comparison between the two sedative regimens’ times (* indicates a *p*-value < 0.0001 between the two groups).

Between 2009 and 2012, a total of 117 ABR procedures were performed using PK, while 73 were performed using CH. Patients’ demographics are summarized in Table 1. The PK group's patients were older than the CH group's patients (Table 1). Patients in the PK group had a lower heart rate at procedure completion compared to the CH group; they also had a significantly higher percentage of transient hypoxemia and a higher percentage of patients receiving supplemental oxygen; however, apnea was not statistically different between the groups (Table 2). No serious adverse events occurred in either group. However, 9 patients (12%) in the CH group failed to achieve a moderate level of sedation and 10 patients (14%) needed a subsequent dose of CH to maintain the sedation. Patients in the PK group had a significantly shorter procedure time, recovery time, and total nurse time (Fig. 1). Adjusting for age and weight did not affect the group comparisons (*p* < 0.0001 for heart rate at procedure completion, procedure time, recovery time, and total nurse time; *p* = 0.0114 for desaturation; *p* = 0.0021 for receiving supplemental oxygen; *p* = 0.27 for apnea).

**Table 1:** Demographics of the two sedative regimen.^a^

	Chloral hydrate (*n* = 73)	Propofol (*n* = 117)	*p*-Value
Age (years)	1.8 (1.0)	2.7 (2.1)	0.003
Weight (kg)	11.3 (2.8)	13.1 (4.8)	0.02
Female	28 (38%)	49 (42%)	0.63

a Data presented as Mean (SD) or *n* (%).

**Table 2:** Comparison between the two sedative regimen dosing and adverse effects.^a^

	Chloral hydrate (*n* = 73)	Propofol (*n* = 117)	*p*-Value
Propofol total dose (mg/kg)	n/a	5.4 (1.9)	n/a
Chloral hydrate dose (mg/kg)	33.4 (7.7)	n/a	n/a
Heat rate pre-sedation	131.4 (18.4)	126.3 (22.7)	0.12
Heart rate after procedure was completed	115.8 (17.2)	101.2 (15.4)	<0.0001
Hypoxemia	1 (1%)	12 (10%)	0.0183
Apnea	0 (0%)	1 (1%)	0.43
Oxygen supplementation	0 (0%)	10 (9%)	0.01
Desired level of sedation achieved	64 (88%)	117 (100%)	<0.0001
A second dose of medication given	10 (14%)	n/a	

a Data presented as Mean (SD) or *n* (%) as appropriate.

## Discussion

The use of ABR under some form of sedation is currently the “gold standard” test to diagnose hearing loss in infants and children who are not developmentally ready or unable to complete behavioral audiometry.^16^, ^17^ While chloral hydrate was widely used as oral sedative hypnotic drug; issues concerning its efficacy and safety continue to arise.^2^ On the other hand, combining propofol and ketamine has been evaluated in large case studies and found to be safe and effective when administered by skilled personnel, resulting in a more rapid recovery, shorter stay and smoother emergence.^18^, ^19^

In our study, 88% of children in the CH group successfully achieved moderate level of sedation using an average dose of 33.4 mg/kg. However, 14% of our patients required more than one dose of CH due to agitation or waking up during the testing. Our data is similar to what Valenzueal et al.^5^ reported where the majority of pediatric patients were successfully sedated using oral CH. Avolnitou et al. also concluded that the vast majority of children were sedated successfully, while 50% of them required a second dose of CH to induce the sedation. Around 12% of children in the CH group had failed to reach the desired level of moderate sedation. All children in the PK group were successfully sedated, which is consistent with the study performed by Akin et al.^3^

In terms of time efficacy, the mean procedure time, recovery time and total nurse time were significantly lower in the PK group compared to the CH group. The procedure time in the CH group is consistent with Avlonitou et al.^2^ findings where the average time for the procedure was about 50 min. The most likely explanation of our findings in terms of time efficacy is the extremely rapid onset and short duration of action of propofol.^20^, ^21^

In terms of adverse events, patients in the PK group had a 10% incidence of transient hypoxemia corrected with regular nasal cannula compared to only 1% in the CH group. Akin et al. reported that transient hypoxemia occurred in 11% of pediatric patients who received propofol for ABR testing. The Pediatric Sedation Research Consortium reported a hypoxia rate around 5% for procedural sedation when propofol was used.^15^ It is possible to explain this partially due to the longer duration and less stimulation during an ABR test compared to other short, more painful procedures, such as a spinal tap or a bone marrow aspiration.

To our knowledge, this is the first study that compares using oral chloral hydrate to intravenous propofol and ketamine for sedation for ABR. Our study shows that procedural deep sedation using a combination of propofol and ketamine for ABR testing is a more efficient regimen than moderate sedation using chloral hydrate with respect to procedure time, recovery time and total nursing time. Deep sedation approach using propofol and ketamine carried a higher incidence of transient hypoxemia compared to the chloral hydrate approach.

Our study has a number of limitations; first, this is a single center retrospective non-randomized study. Second, it compares two different sedation approaches performed by two different teams where there are a number of variables that are impossible to control. The dose of chloral hydrate was used to achieve only moderate sedation in our study and could be the reason for having a higher failure rate and lower adverse events.

## Conclusion

This study demonstrates that both intravenous propofol-ketamine and oral chloral hydrate are effective methods of sedating children undergoing ABR testing in the outpatient setting. However, deep sedation approach using propofol-ketamine is superior in terms of efficiency and offers some workflow advantages over moderate sedation using chloral hydrate. Given the higher incidence of transient hypoxemia compared to chloral hydrate, the use of this sedation strategy should be restricted to practitioners highly trained in the management of the pediatric airway and cardiorespiratory monitoring.

## Conflicts of interest

The authors declare no conflicts of interest.
